# Arabidopsis Lectin EULS3 Is Involved in ABA Signaling in Roots

**DOI:** 10.3389/fpls.2020.00437

**Published:** 2020-04-17

**Authors:** Malgorzata Dubiel, Tom Beeckman, Guy Smagghe, Els J. M. Van Damme

**Affiliations:** ^1^ Laboratory of Biochemistry and Glycobiology, Department of Molecular Biotechnology, Ghent University, Ghent, Belgium; ^2^ Laboratory of Agrozoology, Department of Plants and Crops, Ghent University, Ghent, Belgium; ^3^ Department of Plant Biotechnology and Bioinformatics, Ghent University, Ghent, Belgium; ^4^ VIB-UGent Center for Plant Systems Biology, Ghent, Belgium; ^5^ Center for Advanced Light Microscopy, Ghent University, Ghent, Belgium

**Keywords:** EUL lectin family, abscisic acid, root, stress, CRISPR

## Abstract

The *Arabidopsis thaliana* lectin ArathEULS3 is upregulated in particular stress conditions and upon abscisic acid (ABA) treatment. ABA is a plant hormone important for plant growth and stress responses. During stress ABA is perceived by PYR/PYL/RCAR receptors, inhibiting protein phosphatases PP2Cs thereby enabling SNRK2s kinases to start downstream phosphorylation cascades and signaling. PYL9, one of the ABA receptors was identified as an interacting partner for ArathEULS3. Promoter::GUS activity studies revealed the expression of *ArathEULS3* in the central root cylinder and the cells flanking young lateral root primordia, and showed enhanced expression in root tips after ABA treatment. Transcript levels for *ArathEULS3* increased after exposure to ABA and osmotic treatments. ArathEULS3 CRISPR KO mutants served as a tool to expand the knowledge on the role of ArathEULS3 in plant development. KO lines revealed a longer root system compared to WT plants, and showed reduced sensitivity to ABA, salt, and osmotic conditions. Additionally it was noted that the KO mutants had more emerged lateral roots when grown in high osmotic conditions. Together these data suggest that ArathEULS3 may be an important player in ABA responses in roots.

## Introduction

Plants have developed a sophisticated set of physiological responses, in which the reaction to different environmental conditions is mediated by different hormones. The plant hormone abscisic acid (ABA) acts as a stress hormone important during drought and high salinity conditions as well as during seed maturation (Yoshida et al., 2019). However plants also maintain low concentrations of this hormone even in non-stress conditions. Plant roots are the first to sense changing soil conditions like water shortage or osmotic pressure, which is why ABA levels rise first in the roots. When ABA reaches the leaves, it causes closing of the stomatal aperture, by inducing stress-responsive genes, reducing the entry of CO_2_ to the leaf, and ensuring higher tolerance to dry soil conditions. A breakthrough in understanding the mechanisms behind ABA signaling was made in 2009 when Ma et al. (2009) and Park et al. (2009) discovered that the nucleocytoplasmic ABA receptors (PYR/PYL/RCAR) can inhibit type 2C protein phosphatases (PP2Cs) in the presence of ABA, which in turn release the protein kinases (SNRK2s). Activated SNRK2s phosphorylate ABA-responsive transcription factors, thereby activating ABA-responsive genes.

Lateral root formation in Arabidopsis starts in the elongation zone, where the xylem pole pericycle cells are primed by the phytohormone auxin to become a lateral root. These specifically programmed cells are called pre-branch sites. They retain their stem cell activity and have the potential to become a lateral root (Banda et al., 2019). The initiation of a lateral root takes place in the differentiation zone of the root and causes an asymmetric cell division and formation of a lateral root primordium, a process also promoted by auxin. Since the lateral root primordia are located inside the root and are surrounded by several other cell layers (endodermis, cortex, and epidermis) they need to find a way to pass through the overlying tissues. Auxin is also responsible for signaling through the cell layers in order to change their properties, such as loosening up of the cell walls in order to ensure the emergence of the new roots. However in the roots, auxin is not the only important hormone player—ABA plays an important role in influencing root architecture during drought, osmotic and salt stresses. Suberin is a hydrophobic polymer which is formed on the surface of endodermal cells (Barberon, 2017). The formation of suberin lamellae prevents the water and nutrient uptake at the endodermis level ensuring selectivity towards compounds taken up by the plant and transported through the vascular system to the leaves. In stress conditions like drought or high salinity the suberization is increased through ABA signaling. The ectopic suberin deposition occurs in young root parts and in the cortex (Barberon et al., 2016). Next to suberin deposition, in high salinity environment, ABA can both stimulate primary root growth as well as inhibit lateral root growth, through endodermal signaling. During water deficit ABA also causes an inhibition of lateral root initiation, a phenomenon recently described as xerobranching (Orman-Ligeza et al., 2018). Mutants defective in PYR/PYL ABA receptors turned out to be insensitive to transient ABA treatment and formed lateral roots. Moreover, the lateral root priming might be repressed by ABA accumulation in the basal meristem of the root tip, disrupting the oscillatory auxin responses. Interestingly, auxin plays a pivotal role in the emergence of lateral root in the direction of more water, a process called hydropatterning, which was shown to be ABA-independent (Bao et al., 2014).

Lectins are proteins, composed of one or more lectin domains, which can be linked to other protein domains (such as protein kinase, F-box or glycosyl hydrolase domains), and are able to specifically and reversibly bind to carbohydrate or glycan structures without changing their properties (Van Holle and Van Damme, 2018). Many of the nucleo-cytoplasmic, stress-inducible lectins were shown to be important for both plant development and plant immune responses. For instance, the transcripts of more than 70% of all jacalin-related lectins are upregulated after exposure of the plant to different abiotic and biotic stresses as well as phytohormone treatments, and overexpression of some jacalins resulted in plants with enhanced resistance against pathogens. In contrast to many other lectin families that occur only in some plant families, the *Euonymus europaeus* (EUL) related lectins are present throughout the whole plant kingdom, starting from liverworts to flowering plants, suggesting that these proteins might play a conserved role in plants. The genome of *Arabidopsis thaliana* contains only one EUL gene, further referred to as *ArathEULS3* (Fouquaert et al., 2009). *ArathEULS3* is a stress-responsive protein that locates to the nucleo-cytoplasmic compartment of the plant cell (Van Hove et al., 2011). The expression of *ArathEULS3* is enhanced after hormone treatments (ABA, MeJA, Ethephon) as well as after abiotic (drought, osmotic stress), or biotic (*Pseudomonas syringae*) stresses (Van Hove et al., 2014). In 2014, Li et al. suggested that *ArathEULS3* is involved in ABA signaling through interaction with the ABA receptor PYL9. Furthermore, Van Hove et al. (2015) described the importance of *ArathEULS3* in stomatal closure. Transgenic lines with reduced *ArathEULS3* expression revealed abnormal stomatal closure in the presence of ABA and were reported to be more susceptible to bacteria *Pseudomonas syringae* disease whereas the plants overexpressing EULS3 showed a clear reduction of disease symptoms.

This study aimed to investigate the involvement of *AratEULS3* in ABA signaling in roots. Our analysis confirms that the transcript levels for *ArathEULS3* are upregulated in osmotic stress conditions as well as after exposure to ABA treatment. Promoter::GUS studies suggested that *ArathEULS3* is mostly expressed in the roots. Furthermore CRISPR-induced gene knock-out (KO) lines yielded plants with a larger root system and reduced ABA response, suggesting that ArathEULS3 might be important for ABA signaling.

## Materials and Methods

### Plant Material and Growth Conditions

Wild type *Arabidopsis thaliana* seeds, ecotype Columbia 0, were kindly supplied by Prof. Dr. Richard Strasser (Department of Applied Genetics and Cell Biology, University of Natural Resources and Life Sciences, Vienna, Austria). When grown *in vitro*, seeds were surface sterilized in 70% (v/v) ethanol for 2 min, followed by 8 min in 5% (v/v) NaOCl (Sigma-Aldrich, St Louis, MO, USA). Afterwards, the seeds were rinsed eight times with sterile distilled water. The sterilized Arabidopsis seeds were sown *in vitro* on solid ½ Murashige and Skoog (MS) medium (2.154 g/L MS basal salt [Duchefa Biocheme, Haarlem, The Netherlands], 10 g/L sucrose [Duchefa], 0.1 g/L myo-inositol [Duchefa], 0.5 g/L MES - 2-(N-morpholino)ethanesulfonic acid [Carl Roth, Karlsruhe, Germany], 8 g/L plant tissue culture agar [Duchefa], pH 5.7) supplemented with 10 mg/L phosphinothricin (PPT, Duchefa) or 75 µg/ml kanamycin (Duchefa) as selective agents during selection. Alternatively, Arabidopsis seeds were grown in pots containing commercial soil or individually grown in artificial soil (Jiffy-7, 44 mm Ø). Both plants grown *in vitro* and in soil were first stratified for 3 days at 4°C in the dark and then transferred to a growth chamber at 21°C with a 16/8 h light/dark photoperiod.

### Construction of CRISPR Vectors and Generation of ArathEULS3 Knock-Out Transgenic Lines

CRISPR vectors (pEN-Chimera and pDe-*CAS9*) were kindly supplied by Prof. Dr. Holger Puchta (Botanical Institute II, Karlsruhe Institute of Technology, Karlsruhe, Germany). Design of spacer sequences and cloning was performed according to the protocol provided by Schiml et al. (2016). In short, three 20-nucleotide CRISPR spacer sequences targeting three sites within the *ArathEULS3* coding sequence (**Supplementary File S1**, **Supplementary Figure S1**) were designed using CRISPR-PLANT tool (Xie et al., 2014) and purchased from Invitrogen. In order to obtain entry clones, the 20-nucleotide CRISPR spacer sequences were introduced into pEn-Chimera cut with restriction enzyme *Bbs*I. The customized chimeras were then cloned into pDe-*CAS9* by means of Gateway^®^ LR reaction and expression clones were transformed *via* electroporation to *Agrobacterium tumefaciens* strain C58C1 pMP90. *Arabidopsis thaliana* plants, ecotype Col-0 were transformed using the floral dip method (Clough and Bent, 1998).

### Evaluation of Germinal Mutation

Primary transformants (T1) were selected on ½ MS medium supplemented with 10 mg/L PPT. After 7 days in the growth chamber, putative mutants were transferred to artificial soil—Jiffy-7^®^ for another 11 days. Eighteen-day-old plants were subjected to continuous heat stress for 5 days in a plant cabinet (37°C 16/8 h light/dark photoperiod) and then returned to the growth chamber (22°C 16/8 h light/dark photoperiod) to improve CRISPR efficiency (LeBlanc et al., 2018). The mutations were detected by PCR and sequencing of the fragment flanking the target sequence of *Cas9*. Progeny (T2) seeds were analyzed by PCR for the presence of the *Cas9* gene and sequencing of the fragment flanking the target sequence. Obtained results were analyzed using BioEdit^®^ software for the presence of double peaks and TIDE (tracking of indels by decomposition) (Brinkman et al., 2014) in order to verify the indel frequencies. A homozygous mutation identified in T2 plants was confirmed in the T3 generation by sequencing. Plants recognized as heterozygous were screened for homozygous mutants in the T3 generation. Two *Cas9*-free, homozygous KO lines, one with 1 bp insertion (KO1) and one with 1 bp deletion (KO2) within the *ArathEULS3* coding sequence were selected (Procedure described in more detail in **Supplementary File 1**).

### ArathEULS3 OE Lines

Plants overexpressing ArathEULS3 (lines OE3 and OE4) were generated and screened by Van Hove et al. (2014). The *ArathEULS3* transcript levels in two OE lines were determined for 18-day-old *in vitro* grown seedlings. qRT-PCR analysis revealed approximately 100-fold increase in transcript levels for both lines (**Supplementary Figure S4A**). To confirm the presence of the lectin in the OE lines, crude protein extracts were analyzed by Western Blot analysis, using a polyclonal antibody raised against the EUL domain. Two immunoreactive bands representing the ArathEULS3 protein (35.6 kDa) were clearly detected in protein extracts from plants overexpressing the EUL protein. No signal was detected in extracts from the WT plants, most likely due to the very low lectin content in young plants (**Supplementary Figure S4B**).

### Construction of pArathEULS3:GUS Fusions

A 2,252 bp promoter fragment including the 5′UTR region of *ArathEULS3* gene was amplified and cloned using the Gateway^®^ Technology (Life Technologies, Carlsbad, CA, USA). In a first PCR the promoter region was amplified using Platinum *Pfx* DNA Polymerase (Life Technologies). In the second PCR the *attB* sites were added and the PCR product was ligated in the pJET2.1 vector with the CloneJET PCR Cloning kit according to the manufacturer’s instructions (Life Technologies). The promoter sequence was confirmed by sequencing (LGC Genomics, Berlin, Germany). Equimolar amounts of the *att*B flanked PCR product and the donor vector (pDONR221) were used in an overnight BP recombination reaction with the BP Clonase^®^ II enzyme mix. The obtained entry clones were subsequently recombined with the destination vector pKGWFS7 to create an expression clone which was transformed *via* electroporation to *Agrobacterium tumefaciens* strain C58C1 pMP90. Transgenic plants were made using the floral dip method described above. Transformed seeds were selected on ½ MS medium containing 75 µg/ml kanamycin (Duchefa Biocheme, Haarlem, The Netherlands). Two independent, homozygous, transgenic GUS lines were selected, the seeds were multiplied and T4 generation seeds were used in all experiments.

### Histochemical Analyses for GUS Staining

To check the activity of the *ArathEULS3* promoter, two homozygous GUS lines were germinated and grown in vertical position on ½ MS medium for 0, 3, and 8 days after germination (DAG). Mature siliques, flowers, and fully expanded rosette leaves were detached from 58-day-old-plants. Histochemical staining of β-glucuronidase (GUS) was performed according to Jefferson et al. (1987). In short, harvested plants were first incubated in 90% acetone for 30 min at 4°C. Subsequently, the plants were washed 3 × 5 min in phosphate buffer (0.1 M NaH_2_PO_4_.H_2_O [VWR, Darmstadt, Germany] and 0.1 M Na_2_HPO_4_ [VWR], pH 7.0) and then incubated at 37°C for 30 min in the GUS pre-incubation buffer (phosphate buffer containing 0.5 mM K-ferricyanide and 0.5 mM K-ferrocyanide). Then they were stained in the GUS assay buffer (GUS pre-incubation buffer containing 2 mM 5-bromo-4-chloro-3-indolyl β-D-glucuronic acid [Thermo Scientific, Madison, USA]) and incubated overnight (18 h) at 37°C. The reaction was stopped by washing plants in phosphate buffer, followed by incubation in 70% ethanol for removal of chlorophyll.

### ArathEULS3 Promoter Analyses in Stress Conditions

Two hormones, 10 µM 1-naphthaleneacetic acid (NAA) and 100 µM ABA and two abiotic stresses, salt (150 mM NaCl) and drought (20% w/v PEG6000) were used in stress assays including p*ArathEULS3*::GUS lines and WT plants. ½ MS medium was used as a control for salt and drought treatment. Since the stock solutions for the hormones (ABA and NAA) were made in ethanol, control plants were kept on liquid medium containing 0.001% ethanol. For analyses of *ArathEULS3* expression in stress conditions the WT seeds were germinated and grown *in vitro* on top of a 20 µM nylon mesh overlaid on ½ MS medium. Fourteen-day-old seedlings were subjected to stresses by transferring seedlings on the mesh to new Petri dishes filled with ½ MS liquid medium containing the indicated concentrations of hormones or PEG6000, and incubated at 21°C for 5 or 12 h, collected, and frozen in liquid nitrogen. To assess changes in the expression pattern after stress (ABA, NAA, salt, and drought), 8-day-old seedlings of p*ArathEULS3*::GUS lines, grown vertically on top of a 20 µM nylon mesh overlaid on ½ MS medium were subjected to stress as described above. For each treatment at least 6 seedlings were collected and the GUS histochemical staining was performed.

### Real-Time Quantitative RT-PCR

Total RNA was extracted using TriReagent^®^ (Sigma-Aldrich). DNase I (Thermo Scientific) was used to digest single- and double stranded DNA. RNA concentration and quality was determined with the NanoDrop 2000 spectrophotometer (Thermo Scientific). First-strand cDNA was synthesized from 1 µg of total RNA with oligo(dT)25 primers and 200 U of M-MLV reverse transcriptase (Thermo Scientific) and then diluted 2.5 times. The quality of the cDNA was tested in a standard reverse transcriptase PCR (RT-PCR) using the reference gene Protein phosphatase 2A (PP2A). PCR amplification products were checked on a 1.5% agarose gel. Quantitative real-time PCR (qRT–PCR) was performed with the 96-well CFX Connect™ Real-Time PCR Detection System (Bio-Rad Laboratories, Hercules, CA, USA) using the SensiMix™ SYBR^®^ No-ROX One-Step kit (Bioline Reagents Limited, London, UK). Reactions were conducted in a total volume of 20 μl containing 2 μl cDNA template, 1 × SensiMix™ SYBR^®^ No-ROX One-Step mix, and 500 nM gene specific forward and reverse primer. Three independent biological replicates with each two technical replicates were analyzed, each containing a pool of approximately fifty seedlings. qRT-PCR was performed under following conditions: 10 min at 95°C, 45 cycles of 15 s at 95°C, 25 s at 60°C, and 20 s at 72°C and a melting curve was generated after every qRT-PCR run. All expression data were normalized using three reference genes: PP2A, TIP41 and UBC9 (Czechowski et al., 2005). All melting curves were analyzed after each run (Bio-Rad CFX Manager 3.1 software) and reference gene stability and quality control of the samples were validated in the qBASEPLUS software (Hellemans et al., 2007). Gene specific primers were evaluated by verification of the amplicon size and sequence as well as determination of the amplification efficiency (**Supplementary Table S4**).

### Root Phenotype in Normal and Stress Conditions

*Arabidopsis thaliana* seeds with a gene KO or overexpressing ArathEULS3, were germinated on square Petri dishes (120 ×120 mm). After 3 days of stratification at 4°C, Petri dishes were placed in the growth chamber in the upright (78° angle) position in order to allow the visualization of root growth. To assess whether the stress has an impact on the root growth, 3-day-old seedlings (WT, OE3, OE4, KO1, and KO2) grown *in-vitro* were transferred in septic conditions to a new ½ MS, solid medium supplemented with 10 µM abscisic acid (ABA), salt (150 mM NaCl), or PEG6000 with a water potential of −0.5 MPa corresponding to a concentration of about 12% (w/v). Due to the fact that in the presence of PEG6000 agar used to make plates cannot solidify, the PEG-infusion method described by Van Der Weele et al. (2000) was used. Since the stock solution for ABA was made in ethanol, control plants were kept on ½ MS medium containing 0.001% ethanol. After 10 days in the growth chamber the plates were scanned. The root length and lateral root density of three biological replicates with a minimum of 17 measurements per condition were analyzed from the pictures using the freeware imaging software package Fiji (Schindelin et al., 2012). When grown on medium supplemented with PEG large variations in the number of emerged lateral roots were observed within each genotype. Therefore, a scoring system was developed for the evaluation of lateral root density, based on the number of emerged lateral roots per cm of root at 7 days post transfer onto a medium with stress: score 0, no lateral roots; score 1, lateral root density 0–0.4 roots/cm; score 2, lateral root density 0.41–0.8 roots/cm; score 3, lateral root density 0.81–1.2 roots/cm; score 4, lateral root density >1.2 roots/cm.

### Statistical Analysis

Statistical analysis was conducted using SPSS Statistics 22 (IBM) and the data were considered statistically significant for p < 0.05. The assumption of normality was tested with the Shapiro-Wilkinson test and the equality of variances of normally distributed data was assessed using the Levene’s test. Comparisons between the WT and the transgenic lines were made by using Independent-samples T-test with Bonferroni correction for normally distributed data or Mann-Whitney U test for not normally distributed data. All results for normally distributed data are shown as means ± SE (standard error) or SD (standard deviation) or median values when data were not normally distributed. Statistical analysis for lateral root scoring was performed using the Jonckheere-Terpstra test, which is better suited for ordinal data (p < 0.05, ** p < 0.01, *** p < 0.001). Statistical analysis for qRT-PCR data was evaluated with the REST-384 software using the pair wise fixed reallocation randomization test (with 2,000 randomizations) (Pfaffl et al., 2002).

## Results

### GUS-Reporter Gene Analysis of ArathEULS3 Promoter

**Figure 1:**
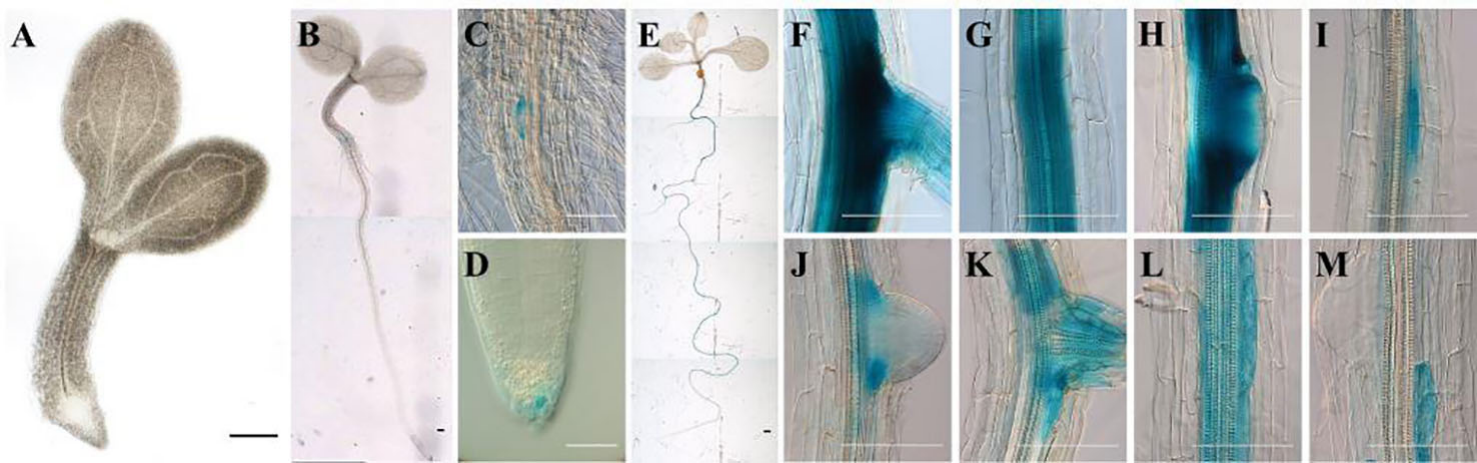
Expression pattern of p*ArathEULS3*::GUS in *Arabidopsis thaliana* seedlings. **(A)** 1-day-old seedlings show no expression, **(B–D)** GUS staining in the root-hypocotyl transition zone and root cap of 3-day-old seedling, **(E–M)** high expression in the roots of 8-day-old plants. Panels **F–M** show successive selected parts moving down the same root. GUS staining was carried out for 18 h. Scale bars represent 0.1 mm for images **C, D** and **E–M**, 1 mm for **A, B, E**.

To determine the tissue specific *ArathEULS3* expression, promoter::GUS studies were performed. A construct comprising 2,252 bp upstream of the translation start was fused to a reporter gene encoding β-glucuronidase (GUS). Homozygous transgenic lines were selected and analyzed for promoter activity throughout plant development using histochemical staining. No expression was observed in very young, 1-day-old seedlings (**Figure 1A**) even after 18 h GUS staining. Promoter activity was observed in the root cap starting from 3 days after germination (DAG) and in the root-hypocotyl transition zone, particularly in the cells flanking the adventitious root primordium (**Figures 1B–D**). Analysis of plantlets at 8 DAG yielded GUS staining only in the roots (**Figure 1E**); mainly in the oldest parts of the root (**Figures 1F–M**). The expression was detected in the central root cylinder in mature parts of the root differentiation zone (**Figures 1F, G**) as well as in cells flanking young lateral root primordia (**Figure 1J**). Patchy staining was observed in the younger part of the root differentiation zone (**Figures 1K–M**). No expression was present in the root meristem, endodermis, cortex, and epidermis. Staining experiments of the aerial plant parts for different plant developmental stages revealed no *ArathEULS3* expression in the leaf tissues (data not shown).

### Stress Regulates the Expression Level and Pattern for ArathEULS3

**Figure 2:**
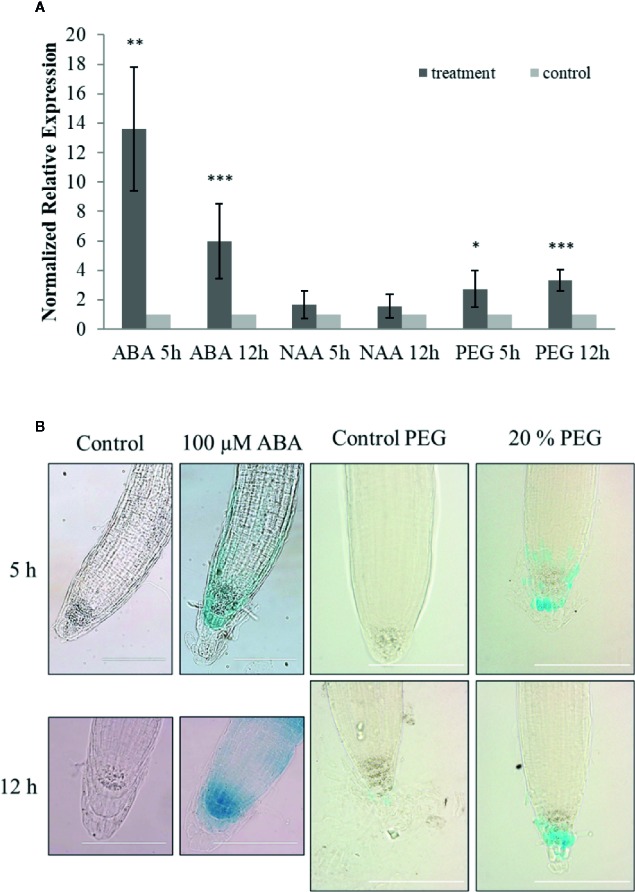
Stress responsiveness of *ArathEULS3* expression. **(A)** Normalized relative expression of *ArathEULS3* in 14-day-old seedlings after ABA, NAA, and PEG treatment. Bars represent means ± SE normalized relative *ArathEULS3* expression compared to mock-treated plants. Asterisks indicate statistically significant differences to the expression level in mock treated seedlings (*p **≤** 0.05, **p **≤** 0.01, ***p **≤** 0.001; REST analysis). The graphs represent the results of three independent biological replicates. **(B)** GUS staining of 8-day-old p*ArathEULS3*::GUS lines treated with ABA and PEG for 5 and 12 h, compared to mock-treated plants. GUS staining was performed for 18 h. GUS staining was observed in more than 90% of the plants treated with ABA. GUS staining was apparent in 70% and 35% of the plants after 5 and 12 h of PEG treatment, respectively. The scale bar represents 0.1 mm.

Expression of *ArathEULS3* was examined in 14-day-old seedlings subjected to ABA, auxin (NAA), and osmotic (20% PEG) stress for 5 and 12 h. Transcript levels for *ArathEULS3* were determined by qRT-PCR analysis (**Figure 2A**). *COR15A* gene was used as a control for ABA treatment (Chen et al., 2009). The qRT-PCR analysis revealed a significant upregulation of *COR15A* in both ABA and PEG treatment (**Supplementary Figure S3**), which indicates that plants sensed the stress treatments. The expression level of *ArathEULS3* was not affected by auxin treatment but both ABA and osmotic stress caused a significant increase in the transcript levels. The strongest effect on *ArathEULS3* expression was observed after 5 h of ABA treatment (13-fold upregulation after 5 h treatment and 5-fold after 12 h treatment). Osmotic stress induced by PEG caused a small, but significant upregulation of about 3 fold both after 5 and 12 h.

To determine the promoter activity in stress conditions 8-day-old p*ArathEULS3*::GUS lines were subjected to ABA, NAA, salt, and drought stress for 5 and 12 h. Overnight GUS staining after ABA treatment revealed increased promoter activity in the root tips of more than 90% of the plants tested (**Figure 2B**). The staining in the root tips was stronger in 70% of the plants subjected to 5 h PEG treatment and 35% of the plants after 12 h PEG. No clear differences in staining patterns were observed for plants treated with auxin or salt.

### ArathEULS3 KO Mutants Show a Reduced Inhibition of Primary Root Growth and Lateral Root Formation in Normal and Osmotic Stress Conditions

**Figure 3:**
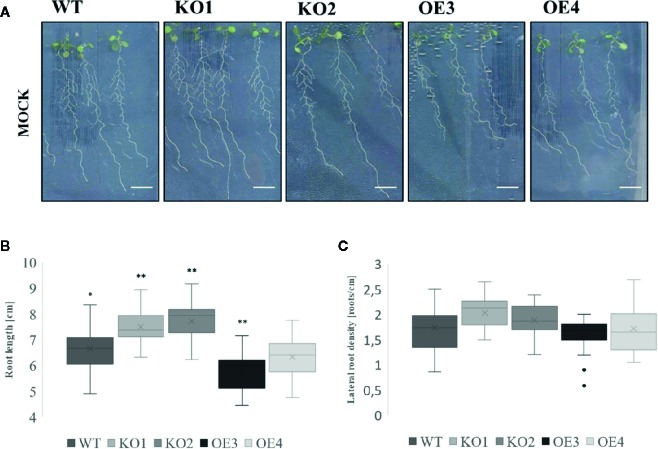
Growth of transgenic lines under normal growth conditions. **(A)** Root phenotype, **(B)** root length, **(C)** and lateral root density of 10 day-old transgenic lines compared to WT. The graphs represent three biological replicates with minimum 17 data points per condition. The comparison between the root length of WT and transgenic lines was made by using Independent-samples T-test for normally distributed data. The comparison of lateral root density of different transgenic lines to WT was made by using Mann-Whitney test for not normally distributed data. The distributions of root length and lateral root density values are shown as box and whisker plots. The boxes represent the 25–75th percentiles, the median is indicated as a horizontal line, the mean is indicated as a cross, the outliers are represented as circles. The whiskers are equal to 1.5× the interquartile range (*p < 0.05, **p < 0.01).

To investigate the role of ArathEULS3 in plant responses to salt and osmotic stress the root growth phenotype of two KO lines and two OE lines was examined in the presence of salt (150 mM NaCl), drought (PEG), and ABA (10 µM). The construction and selection of ArathEULS3 KO lines is described in detail in the **Supplementary File S1**. The growth of ArathEULS3 KO and overexpression lines was analyzed in non-stressed conditions (mock treatment). The roots of both KO lines were significantly longer than the roots of WT plants, whereas the root length of one of the OE lines was significantly shorter (**Figure 3**). The lateral root density did not differ in any of the mutant lines. No significant differences were observed in different mock treatments (**Supplementary Figures S5** and **S6**).

**Figure 4:**
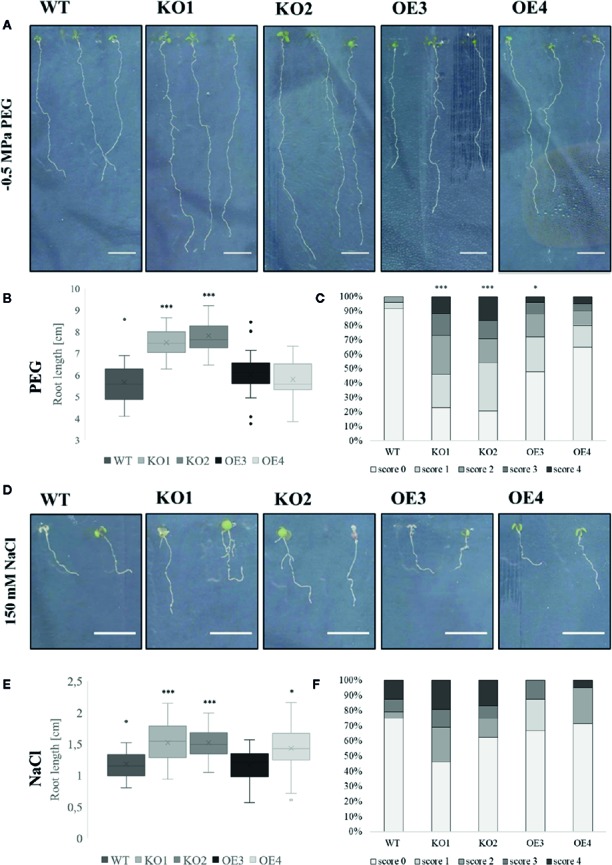
Growth of transgenic lines after salt and osmotic stress treatments. **(A**, **D)** Root phenotype, **(B**, **E)** root length and **(C**, **F)** lateral root density of WT and transgenic lines grown on medium with or without PEG (−0.5 MPa) or salt (150 mM). The graphs represent three biological replicates with minimum 17 data points per condition. Scoring system for evaluation of lateral root density is explained in the *Materials and Methods*. The seeds of WT plants and transgenic lines (KO1, KO2, OE3, and OE4) were germinated *in vitro* for 3 days and the young seedlings were then transferred onto a medium containing stress factors (150 mM NaCl or −0.5 MPa PEG6000) for another 7 days. The comparison between the root length of WT and transgenic lines was made by using Independent-samples T-test for normally distributed data. The distributions of root length values are shown as box and whisker plots. The boxes represent the 25–75th percentiles, the median is indicated as a horizontal line, the mean is indicated as a cross, the outliers are represented as circles. The whiskers are equal to 1.5× the interquartile range. The comparison between the scoring of lateral root density between WT and transgenic lines was made by using Jonckheere-Terpstra test. (*p < 0.05, ***p < 0.001).

**Figure 5:**
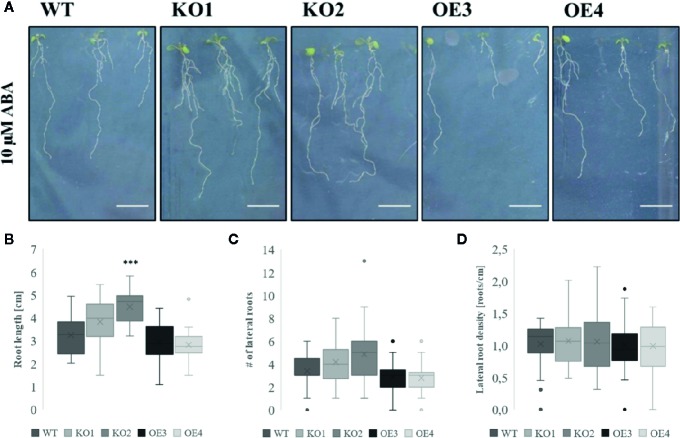
Growth of transgenic lines after ABA treatment. **(A)** Root phenotype and **(B)** root length of WT and transgenic lines grown in the presence or absence of 10 µM ABA. **(C)** Number of lateral roots and **(D)** lateral root density in plants grown on medium supplemented with ABA. The graphs represent three biological replicates with minimum 17 data points per condition. The seeds of WT plants and transgenic lines (KO1, KO2, OE3, and OE4) were germinated *in vitro* for 3 days and the seedlings were transferred onto a medium containing 10 µM ABA for another 7 days. The comparison between the root length of WT and transgenic lines was made by using Independent-samples T-test for normally distributed data. The statistical analysis for the number of lateral roots and lateral root density was performed using Mann-Whitney U test for not normally distributed data. The distributions of root length, number of lateral roots, and lateral root density values are shown as box and whisker plots. The boxes represent the 25–75th percentiles, the median is indicated as a horizontal line, the mean is indicated as a cross, the outliers are represented as circles. The whiskers are equal to 1.5× the interquartile range (***p < 0.001).

After treatment with PEG and salt both KO lines revealed a longer primary root compared to WT plants (**Figures 4A, B, D, E**). However, after treatment with ABA only the roots of KO2 were significantly longer compared to roots of WT plants (**Figures 5A, B**). When grown in osmotic (PEG) stress conditions both KO mutants had a substantially higher number of emerged lateral roots compared to WT plants (**Figure 4C**). Fewer differences were observed in lateral root density for plants grown on high salinity medium (**Figure 4F**). Since ABA is known to mediate the inhibition of lateral root emergence in osmotic stress conditions (Deak and Malamy, 2005), the changes in lateral root number and the lateral root density were examined after exposure to 10 µM ABA concentrations. Neither the lateral root number nor the lateral root density in the transgenic lines differed significantly from the WT (**Figures 5C, D**). Taken together, these results suggest that ArathEULS3 KO lines revealed a root phenotype which is characterized by a faster root growth. Roots of KO lines are less sensitive to osmotic stress compared to WT plants.

Since the root length of ArathEULS3 KO lines is larger than the root length of the WT plants under both unstressed and stressed conditions, the differences in root length between mock treatments and different stress conditions, including ABA, PEG, and salt treatments, were quantified. This analysis revealed that compared to non-stress conditions the average difference in root lengths between KO and WT plants grown in the presence of stress was considerably larger, irrespective of the stress factor used (**Table 1**).

**Table 1:** Differences in root length for WT plants and KO lines grown in the presence or absence of a stress factor.

	Root length difference	
	KO1 compared to WT	KO2 compared to WT
MOCK (+0.001% EtOH)	8.5%	13.3%
10 µM ABA	17.7%	38.0%
MOCK	12.7%	16.0%
−0.5 MPa PEG	32.3%	37.9%
150 mM NaCl	28.9%	28.9%

## Discussion

**Figure 6:**
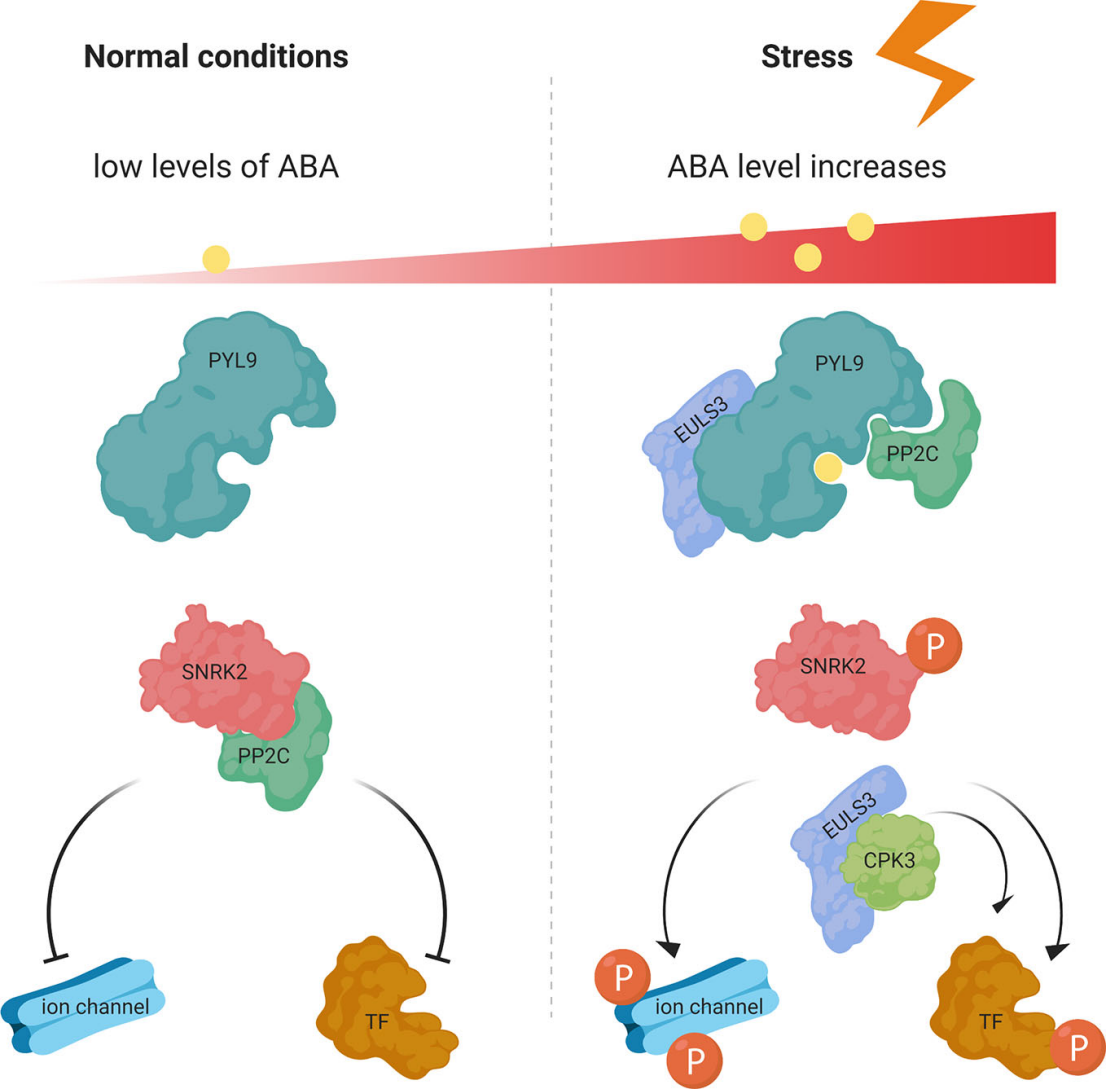
ABA-mediated signaling pathway in normal and stress conditions. At low ABA concentrations in normal conditions the SnRK2 protein kinase activity is inactivated by PP2C phosphatases. Under stress conditions ABA binds to PYR/PYL/RCARs, which binds and inhibits the P2C phosphatases, releasing the SnRK2 kinases, which then phosphorylate downstream targets and trigger ABA-induced physiological responses. ArathEULS3 (EULS3) is known to interact with PYL9 and CPK3. TF, transcription factor; P, phosphorylation. Figure adapted and re-drawn from (Ullah et al., 2017).

ABA is an important phytohormone which in non-stress conditions is maintained in the plant at low levels. The basal ABA concentration is important for primary plant metabolism, supports plant growth and development, and has an inhibitory effect on leaf emergence and promotes root growth (Yoshida et al., 2019). PEG is a high molecular weight solute which can mimic drought stress (Alqurashi et al., 2018) and ABA is one of the most important transducer of drought, salt, and osmotic stresses (Harris, 2015). Our results suggest that exposure of WT plants to ABA and PEG results in an increased mRNA level for *ArathEULS3* in the WT plants. Rasheed et al. (2016) also reported *ArathEUSL3* as one of the highly up-regulated genes during progressive drought stress. The mRNA levels were 4-fold upregulated in the roots already at 3 days after ceasing the watering and increased after further exposure to drought stress to 5.5-, 8.1-, and 12.6-fold up-regulation after 3, 5, and 7 days, respectively. Interestingly the significant upregulation of *ArathEULS3* mRNA levels in the shoots was only observed after 7 and 9 days (4- and 7.3-fold, respectively). Additionally the gene expression analysis after osmotic stress (300 mM mannitol) reported by Kilian et al. (2007) revealed changes in *ArathEULS3* mRNA levels both in the roots (2.8-fold after 1 h of treatment, up to 8.8-fold after 24 h) and in the shoots (5.5-fold after 3 h, up to 22-fold after 24 h). Similar, but slightly milder effects were observed after salt treatment (150 mM NaCl), with a maximum of 5.4-fold upregulation in the roots after 6 h and in the shoots after 24 h of treatment (13.12-fold upregulation). Moreover, several other recent publications linked ArathEULS3 to ABA signaling. ArathEULS3 was shown to interact with CPK3 (**Figure 6**), a calcium-dependent protein kinase (Berendzen et al., 2012) which has an important function in the guard cells and ABA signaling. Pandey et al. (2010) recognized *CPK3* as an ABA-regulated gene with 6-fold upregulation in guard cells and leaves after 3 h of 50 µM ABA treatment on 5-week-old Arabidopsis leaves. Similarly, AratEULS3 mRNA levels were 5.6-fold upregulated in Arabidopsis cell suspension cultures after 20 h of 50 µM ABA treatment (Böhmer and Schroeder, 2011). Furthermore, Wang et al. (2011) listed *ArathEULS3* among the 67 most important ABA-induced genes, specifically in stomata. Arabidopsis overexpressing ArathEULS3 showed fewer disease symptoms after infection with *P. syringae* (Van Hove et al., 2015) and revealed increased tolerance to drought stress (Li et al., 2014). Additionally, the *in-silico* promoter analysis confirmed the presence of many cis-regulatory elements associated with drought/salt and ABA response in the promoter region upstream from the *ArathEULS3* gene (Van Hove et al., 2014).

Our promoter activity analysis with the GUS reporter system did not reveal any staining in leaf tissues for any of the tested developmental stages, not even after any of the stress treatments. GUS staining was mostly detected in the roots; especially in the central cylinder of the differentiation zone and in the cells flanking young lateral root primordia. However, the identification of the cell wall proteome revealed the presence of EUL peptides in *A. thaliana* leaves (Hervé et al., 2016). The lack of GUS expression in leaves in our experiments might be explained by the promoter region used for the analysis. It is well-established that transcription regulatory elements are present not only in the promoter sequence preceding the gene sequence but are also located within intron sequences or distal enhancers which can be located even thousands of nucleotides up- or downstream from the coding sequence (Le Hir et al., 2003; Bulger and Groudine, 2011). The absence of such regulatory sequences might explain the lack of promoter activity in the leaves in our experiment, though transcripts for *ArathEULS3* have been detected multiple times before in leaves collected at different developmental stages (Wang et al., 2011; Van Hove et al., 2014). After exposure of plants expressing p*ArathEULS3*::GUS to ABA an increase of GUS expression in the root tips, more specifically in the root cap, was apparent, which is reminiscent of the expression pattern obtained using an abscisic acid responsive element (ABRE)-based synthetic promoter (Fujita et al., 2013). Together with the evidence from previous studies (Kilian et al., 2007; Böhmer and Schroeder, 2011; Wang et al., 2011; Van Hove et al., 2014; Rasheed et al., 2016) our results indicate that *ArathEULS3* is definitely an ABA-responsive gene.

Since ArathEULS3 is the only representative of the EUL family in the model plant *Arabidopsis thaliana*, functional redundancy is excluded. This makes Arabidopsis a good candidate for studying the physiological importance of EUL proteins in plants. In our analyses we used two independent, homozygous OE lines and two homozygous KO lines. The use of two mutant lines may be a shortcoming of this study, since it was not always sufficient to draw straight forward conclusions. Our root phenotype analysis revealed that the root architecture of CRISPR ArathEULS3 KO lines differed significantly from the WT plants. Both KO lines yielded a larger root system in normal growth conditions, and one of the ArathEULS3 overexpression lines had significantly shorter roots. ABA regulates the primary and lateral root growth in response to drought or high salinity. Low concentrations of ABA promote the root growth whereas high concentrations inhibit both the primary and the lateral root growth by inhibiting cell division in the meristem (Harris, 2015). Plants defective in ABA biosynthesis are characterized by a larger root system and impaired inhibition of lateral root elongation. ArathEULS3 was reported to interact with the ABA receptor PYL9 (**Figure 6**), one of the most important players in ABA signaling (Li et al., 2014). Similar to ArathEULS3, the expression of PYL9 was most dominant in the vascular root tissues (Antoni et al., 2013). Together with *PYL8*, *PYL9* is the most strongly induced gene of this family after ABA treatment (Tischer et al., 2017), and both *PYL8* and *PYL9* were shown to be important for regulating root architecture (Xing et al., 2016). Interestingly, when overexpressing the *PYL9* gene the authors observed a reduced number of lateral roots and a shorter primary root. The double mutant *pyl8*-*pyl9* revealed a longer root when grown on medium supplemented with ABA resulting in a reduced sensitivity to ABA-mediated primary root growth inhibition and a slightly increased number of lateral roots (Xing et al., 2016), suggesting that *PYL8* and *PYL9* mediate the ability of ABA to inhibit primary root growth. In our analyses, only the ArathEULS3 KO2 line showed a significant increase in the root length after ABA treatment. However the root length of both KO lines was increased in osmotic stress conditions (salt, PEG) compared to WT plants. These data together with the results showing the increase of *ArathEULS3* expression in the root tips upon ABA treatment suggest that *ArathEULS3* could be important for the inhibition of cell division or cell elongation controlled by the ABA-signaling in the root cap in osmotic stress conditions.

Auxin plays a pivotal role in the coordination of lateral root primordial formation and lateral root growth (Vilches-Barro and Maizel, 2015). Interestingly, PYL9, was shown to be a node between the ABA and auxin crosstalk in the roots, through direct interaction with MYB77 and MYB44 transcription factors, regulating their transcriptional activity (Xing et al., 2016). However, *ArathEULS3* expression was not influenced by the presence of high auxin concentrations in the environment. The analysis presented here was performed using whole seedlings. Therefore if the response was specific for one tissue type, it could have been overlooked. In that respect, the analysis done by Bargmann et al. (2013) where gene expression was measured locally in the xylem pole pericycle cells, stele, columella, epidermis, and lateral root cap, showed significant down-regulation of *ArathEULS3* after 3 h of auxin (5 µM IAA) treatment specifically in the xylem pole pericycle cells as well as in the stele, endodermis, and the lateral root cap. This is in agreement with the hypothesis suggesting that *ArathEULS3*, like *PYL9*, could play a role in the interplay of ABA and auxin signaling in roots.

*PYL9* OE lines were characterized by a reduced number of lateral roots (Xing et al., 2016). None of the tested transgenic lines differed in the lateral root number and density from the WT roots under normal growth conditions. It is known that high concentrations of PEG and salt inhibit lateral root emergence. However, our data show that after PEG and salt treatment some of the KO plants clearly had a higher number of emerged lateral roots. Therefore the lateral root density was scored in each plant, which revealed that after transfer to PEG, both KO mutants had a substantially higher number of emerged lateral roots than WT plants. Differences were also observed after salt treatment, however, not statistically significant. Together with specific expression of *ArathEULS3* in the cells flanking lateral root primordia, these data suggest that *ArathEULS3* might play a role in lateral root emergence during stress conditions like water deficit or high salinity. However, this should be confirmed by performing more detailed analyses of cell division in the lateral root primordia and emergence of lateral roots in transgenic lines of ArathEULS3.

Here we present a new set of data suggesting a strong link between ArathEULS3 and ABA responses in roots. The promoter activity assays suggest that *ArathEULS3* expression mainly takes place in the root tissues. Furthermore, the KO lines revealed a root phenotype similar to the double *pyl8-pyl9* mutant, a KO line for the major ABA receptors after osmotic stress and ABA treatment.

## Data Availability Statement

All datasets generated for this study are included in the article/**Supplementary Material**.

## Author Contributions

MD, TB, and ED outlined and designed the study. MD performed the experiments. MD analyzed and interpreted the data. MD and ED prepared the manuscript. ED, TB, and GS conceived and supervised the experiments and critically revised the manuscript. All authors have read, revised, and approved the final manuscript.

## Funding

This research was funded by the Research Council of Ghent University (project BOF15/GOA/005).

## Conflict of Interest

The authors declare that the research was conducted in the absence of any commercial or financial relationships that could be construed as a potential conflict of interest.
