# Supplementary material for: Arabidopsis Lectin EULS3 Is Involved in ABA Signaling in Roots
Source: Front Plant Sci. 2020 Apr 17;11:437. doi: 10.3389/fpls.2020.00437 (PMC7181964; doi:10.3389/fpls.2020.00437)
Supplement: Supplementary file 1 [file DataSheet_1.docx]

Supplementary Methods

### Construction of CRISPR vectors and generation of ArathEULS3 knock-out transgenic lines

CRISPR vectors (pEN-Chimera and pDe-CAS9) were kindly supplied by Prof. dr. Holger Puchta (Botanical Institute II, Karlsruhe Institute of Technology, Karlsruhe, Germany). Design of spacer sequences and cloning was performed according to the protocol provided by Schiml et al. (2016). Three 20-nucleotide CRISPR spacer sequences targeting three sites within the ArathEULS3 coding sequence referred to as CRISPR1, CRISPR2 and CRISPR3 (Supplementary Figure S1) were designed using CRISPR-PLANT tool (Xie et al., 2014). The expression clones were transformed via electroporation to *Agrobacterium tumefaciens* strain C58C1 pMP90. *Arabidopsis thaliana* plants, ecotype Col-0 were transformed using the floral dip method (Clough and Bent, 1998).

Primary transformants (T1) were selected on ½ MS medium supplemented with 10 mg/L PPT. After 7 days in the growth chamber, putative mutants were transferred to artificial soil – Jiffy-7® for another 11 days. 18-day-old plants were subjected to continuous heat stress for 5 days in a plant cabinet (37 °C 16/8 h light/dark photoperiod) and then moved back to the growth chamber (22 °C 16/8 h light/dark photoperiod). Total genomic DNA (gDNA) was extracted from rosette leaves using the slightly adapted Edwards protocol. The integration of the T-DNA was checked by PCR on gDNA using primers which amplify a 501 bp fragment within the Cas9 gene (p688 and p689, Supplementary Table S1) with the following PCR program: 10’ – 95 °C, 35x (30’’ – 95 °C, 30’’ – 54 °C, 45’’ – 72 °C), 5’ – 72 °C. The region around the target mutation in samples containing Cas9 gene was amplified using p690 and p691 primers for CRISPR1 and p694 and p695 for CRISPR2 (Supplementary Table S1) using the same PCR program. Amplicons of the latter PCR were sequenced with primers p692 and p696 (Supplementary Table S1) for CRISPR1 and CRISPR2, respectively (LGC Genomics GmbH 12459 Berlin-Germany). Obtained results were analyzed using BioEdit® software site in order to confirm the activity of the Cas9 enzyme in the first generation (T1). Progeny (T2) seeds were germinated *in vitro* for 1.5 weeks and grown individually in artificial soil (Jiffy-7, 44 mm Ø). Cas9-free mutants were selected by means of PCR as mentioned above. The region around the mutation in those plants was amplified and sequenced (LGC Genomics). Obtained results were analyzed using BioEdit® software for the presence of double peaks and TIDE (tracking of indels by decomposition) (Brinkman et al., 2014) in order to verify the indel frequencies. Homozygous mutation identified in T2 plants was confirmed in the T3 generation by sequencing gDNA extracted from 5 plants. Plants recognized as heterozygous were screened for homozygous mutants in the T3 generation.

### Construction of pArathEULS3:GUS fusions

The promoter sequence of ArathEULS3 gene was cloned using the Gateway® Technology (Life Technologies, Carlsbad, CA, USA). In the first PCR reaction a fragment bigger than the promoter region was amplified using Platinum *Pfx* DNA Polymerase (Life Technologies) using primers p395 and p396 (Supplementary Table S1) and the following conditions: 96 °C – 7’, 40 x (96 °C – 30’’, 54 °C – 30’’, 66 °C – 3’) – 66 °C – 10’’. The product with a correct size was extracted from the gel using Qiaquick gel extraction kit (Hilden, Germany) and used as a template to amplify an 5’ upstream fragment of 2,252 bp including the 5’UTR region of ArathEULS3 gene. In the second PCR the *attB* sites were added using the following program: 96 °C – 7’, 40 x (96 °C – 30’’, 47 °C – 30’’, 66 °C – 3’) – 66 °C – 10’’. The PCR product of a correct size was extracted from the gel and was ligated in the pJET2.1 vector with the CloneJET PCR Cloning kit according to the manufacturer’s instructions (Life Technologies) and the promoter sequence was confirmed by sequencing with primers p425 and p426 (LGC Genomics). The obtained by BP Gateway® reaction entry clones were subsequently recombined with the destination vector pKGWFS7 to create an expression clone which was transformed via electroporation to *Agrobacterium tumefaciens* strain C58C1 pMP90. The transgenic plants were made using the floral dip method described above. Transformed seeds were selected on ½ MS medium containing 75 µg/ml kanamycin (Duchefa). Green seedlings were transferred to soil and then the integration of the T-DNA was checked by PCR using gDNA as a template and kanamycin-specific primers (evd261 and evd463) with the following PCR program: 94 °C – 10', 45 x (94 °C – 30’’, 48 °C – 30’’, 1’ 72 °C) – 72 °C – 5’. Two independent, homozygous, transgenic GUS lines were selected (G1C and G1D), the seeds were multiplied and T4 generation seeds were used in all experiments.

Supplementary Results

### Heat-stress increases efficiency of targeted mutagenesis by CRISPR/Cas9

To construct the ArathEULS3 KO (knockout) lines, the pEN Chimera vector carrying the chimeric sgRNA sequence was recombined with pDE-CAS9 vector harboring *Staphylococcus pyogenes* Cas9 driven under the control of Ubiquitin promoter from parsley and the PPT selection cassette. After transformation of *Arabidopsis thaliana* plants with CRISPR vectors via *Agrobacterium*-mediated floral dip, T1 plants were selected on ½ MS medium supplemented with PPT. The mutation regions of all plants that contained Cas9 gene were sequenced. In the first selection, in total twenty seven T1 plants (15 CRISPR1 and 12 CRISPR2) containing the Cas9 cassette were screened for mutations. Only three plants (20 %) of CRISPR1 and none of CRISPR2 lines showed double peaks on the sequencing chromatogram suggesting that no mutations were introduced. In total, 261 T2 plants were analyzed, of which mutation regions of gDNA samples from 35 Cas9 negative and 7 Cas9 positive plants were sequenced. All tested samples contained WT sequences, which indicated that no heritable mutations occurred in the T1 generation. Moreover in all tested T2 plants still containing the Cas9 gene, the nuclease was not active (no double peaks observed).

Since Cas9 used in this study comes from a bacterial organism (*Streptococcus pyogenes*), with an optimal growth temperature of 40°C, there is a possibility that Cas9 enzyme efficiency might be lower at 21°C – the temperature used for growing Arabidopsis plants. After selection on medium containing PPT T1 plants were subjected to heat stress. Eighteen CRISPR1 and ten CRISPR2 plants containing the Cas9 gene were selected on ½ MS medium supplemented with PPT. After 7 days, the putative mutants were transferred individually to artificial soil for 11 days (soil acclimation) and then subjected to 5 days of continuous heat treatment (37°C). Afterwards the plants were grown continuously at 21°C. Genomic DNA was extracted from rosette leaves of 28-day-old plants and the absence of Cas9 was confirmed by PCR with gene-specific primers targeting a sequence within the Cas9 gene. The mutation regions were amplified and sequenced. The analysis revealed that 39 % of CRISPR1 samples showed double peaks on the sequencing chromatogram. However none of CRISPR2 plants revealed double peaks after sequencing. 22 % of all CRISPR1 sequences screened (coming from plants C1.7, C1.9, C1.13, and C1.21) were different from the one of the WT, which indicates high efficiency of the Cas9 enzyme. The remaining 17% of sequences (coming from plants C1.1, C1.3, and C1.5) were identical to the WT sequence, meaning that the efficiency of Cas9 was most likely lower in those samples.

### Selection of homozygous ArathEULS3 knock-out mutants

Promising CRISPR1 mutants (C1.1, C1.3, C1.5, C1.7, C1.9, C1.13, and C1.21) were grown until maturity and the seeds were collected. In total 136 plants from five transgenic lines were screened for the absence of Cas9 gene. The analysis revealed that only three (C1.3, C1.9 and C1.13) out of five mutant lines segregated in a Mendelian fashion (Supplementary Table 1) and only those plants were used in the following analyses. The segregation ratios in two remaining lines (C1.7 and C1.21) can be explained as Mendelian inheritance of more than one independent loci. If more than one T-DNA is integrated in the genome, plants will segregate in a different manner (two insertions will give 15:1 segregation, three – 63:1 and so on).

Supplementary Table S1 Segregation of Cas9 gene in T2 generation CRISPR mutants. Plants segregating in Mendelian fashion are shown in bold

| Mother T1 plant | No of T2 plants tested | Cas9 (+) | Cas9 (-) |
| --- | --- | --- | --- |
| C1.3 | 17 | 82 % | 18 % |
| C1.7 | 16 | 100 % | 0 % |
| C1.9 | 62 | 69 % | 31 % |
| C1.13 | 32 | 84 % | 16 % |
| C1.21 | 9 | 100 % | 0 % |

After amplification of the target mutation region, twenty-four Cas9-free samples were sequenced in order to confirm the presence of mutations and plant genotype. Sequencing results were analyzed by observation of double peaks at the cleavage site on the sequencing chromatogram, multiple sequence alignment and analysis of indel sizes and types with TIDE (tracking of indels by decomposition) analysis. Mutations were found in 56 % and 60 % of C1.9 and C1.13 progeny plants (T2) respectively, but in none of C1.3 progeny plants, giving an overall efficiency of 38 % in the T2 generation. Plant genotypes can be found in the Supplementary Table 2. According to TIDE analysis, the most commonly occurring mutation in the C1.9 progeny was one bp insertion (including one homozygous plant C1.9-C, KO-1) with two exceptions; in one heterozygous monoallelic plant a seven bp deletion was found (C1.9-H), and one biallelic plant which showed a one bp insertion and one bp deletion (C1.9-D). The progeny of the C1.13 plant showed mostly mutations of three or six bp deletions.

When searching for plants with a gene KO for ArathEULS3, plants with insertions or deletions of 3n nucleotides are not considered interesting due to the insertion or deletion of whole codon sequences, causing insertions or deletions of full amino acids, but not the desired frameshift. Therefore, after transcription and translation of such sequence, there is still a possibility that a protein might fold correctly and retain its function.

Supplementary Table S2 Genotypes found in T2 generation of Cas9-free plants

|  | Wild Type | Heterozygous  monoallelic | Heterozygous  biallelic | Homozygous |
| --- | --- | --- | --- | --- |
| C1.3 | 100 % | 0 % | 0 % | 0 % |
| C1.9 | 44 % | 44 % | 6 % | 6 % |
| C1.13 | 40 % | 20 % | 40 % | 0 % |

The homozygosity of the C1.9-C (KO1) mutant (1 bp insertion) was confirmed in the T3 generation by sequencing the target mutation region from five independent plants, the absence of double peaks on the chromatogram and additional TIDE analysis. Another homozygous mutant with a 1 bp deletion was selected by sequencing DNA of the biallelic C1.9-D progeny plants (Supplementary Figure S2). Out of seven sequenced samples, four were homozygous with one bp insertion, one was again biallelic and two were homozygous with one bp deletion (C1.9-D-6 and C1.9-D-7, KO2). Taken together those results suggest that by using heat stress we managed to improve the efficiency of the CRISPR mediated gene KO and successfully select two independent homozygous ArathEULS3 KO lines.


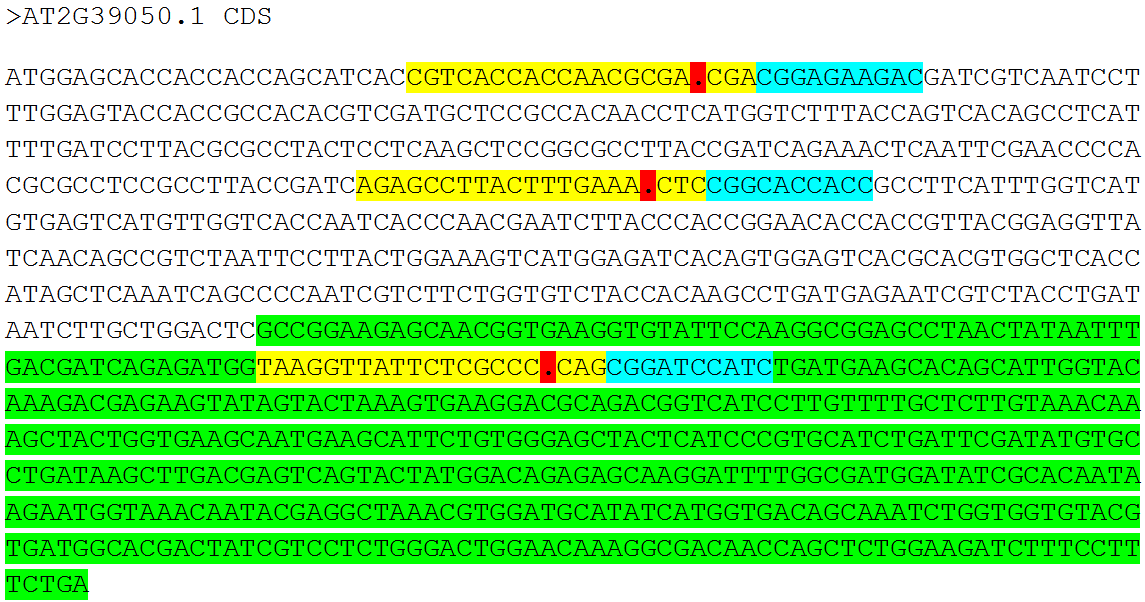


Supplementary Figure S1: CDS of ArathEULS3. Three 20-nucleotide CRISPR spacer sequences (CRISPR1, CRISPR2 and CRISPR3 are indicated in yellow). Protospacer adjacent motifs (PAM) are shown in blue, predicted cleavage sites in red. The EUL lectin domain is highlighted in green.


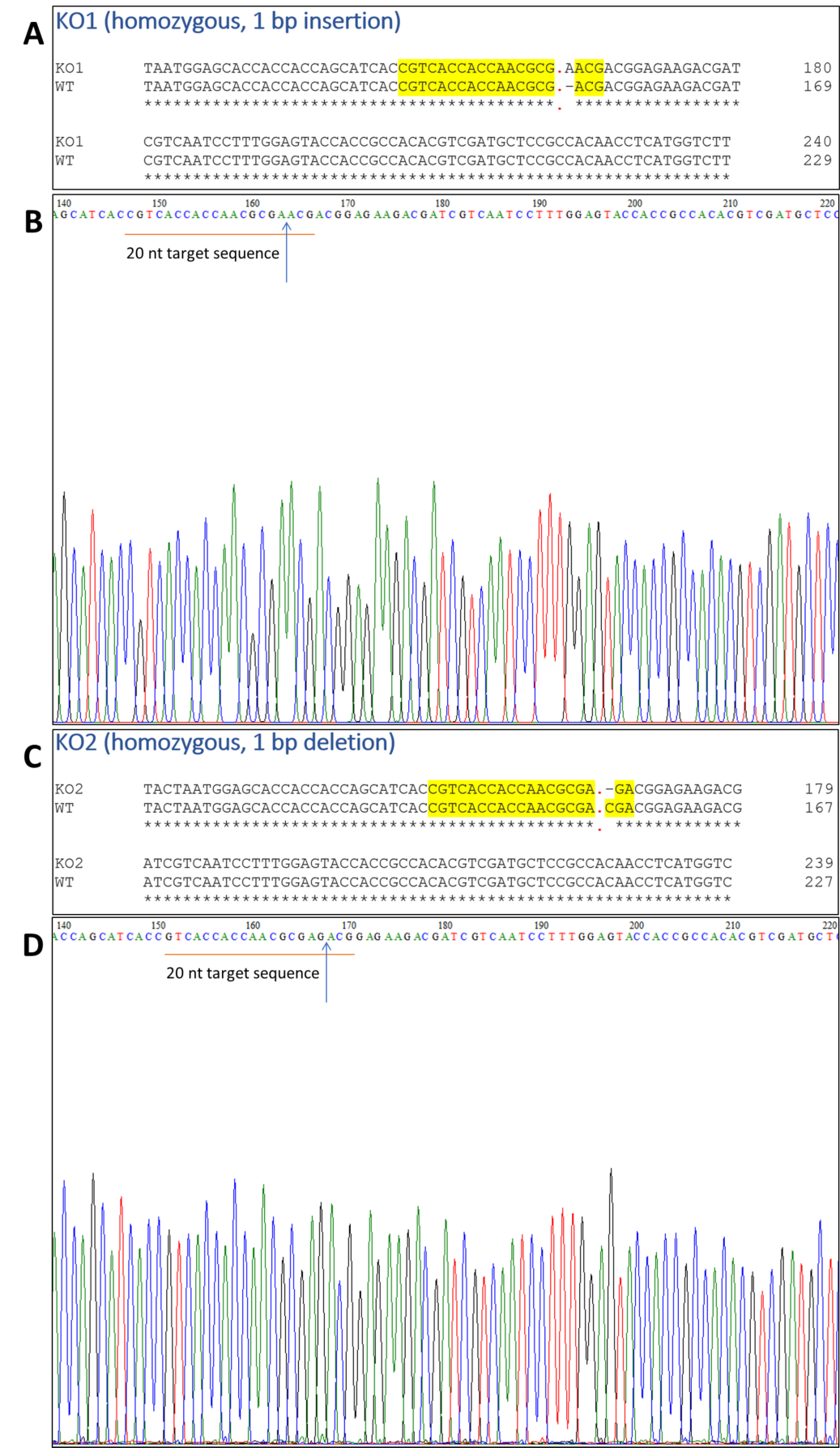


Supplementary Figure S2: Results of selection of ArathEULS3 KO lines. Figures (A) and (C) represent sequence alignments of the ArathEULS3 gene sequence from WT plants with ArathEULS3 gene sequences from KO1 and KO2 mutants. 20 nucleotide target sequences are shown in yellow and predicted cleavage sites in red. Figures (B) and (D) represent sequencing chromatograms of ArathEULS3 gene sequences from KO1 and KO2 mutants, respectively.

References

Brinkman, E. K., Chen, T., Amendola, M., and van Steensel, B. (2014). Easy quantitative assessment of genome editing by sequence trace decomposition. *Nucleic Acids Res.* 42, e168. doi:10.1093/nar/gku936.

Clough, S. J., and Bent, A. F. (1998). Floral dip: A simplified method for Agrobacterium-mediated transformation of *Arabidopsis thaliana*. *Plant J.* 16, 735–743. doi:10.1046/j.1365-313X.1998.00343.x.

Schiml, S., Fauser, F., and Puchta, H. (2016). “CRISPR/Cas-Mediated Site-Specifi c Mutagenesis in *Arabidopsis thaliana* Using Cas9 Nucleases and Paired Nickases,” in *Methods in Molecular Biology* 1469, 110–122. doi: 10.1007/978-1-4939-4931-1_8.

Xie, K., Zhang, J., and Yang, Y. (2014). Genome-wide prediction of highly specific guide RNA spacers for CRISPR–Cas9-mediated genome editing in model plants and major crops. *Mol. Plant* 7, 923–926. doi:10.1093/mp/ssu009.
