# Supplementary material for: Arabidopsis Lectin EULS3 Is Involved in ABA Signaling in Roots
Source: Front Plant Sci. 2020 Apr 17;11:437. doi: 10.3389/fpls.2020.00437 (PMC7181964; doi:10.3389/fpls.2020.00437)
Supplement: Supplementary file 2 [file DataSheet_2.docx]

Supplementary Material

# Supplementary Figures


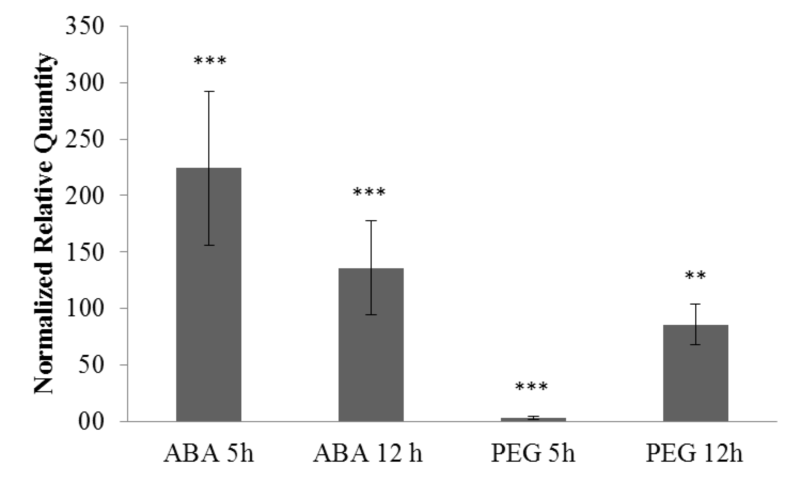


Supplementary Figure S3: Normalized relative expression of COR15A gene after ABA and PEG treatment. Bars represent means ±SE normalized relative AathEULS3 expression compared to mock treated plants. Asterisks indicate statistically significant differences to the expression level in mock treated seedlings (** p≤0.01, *** p≤0.001; REST analysis). The graphs represent the results of three independent biological replicates.


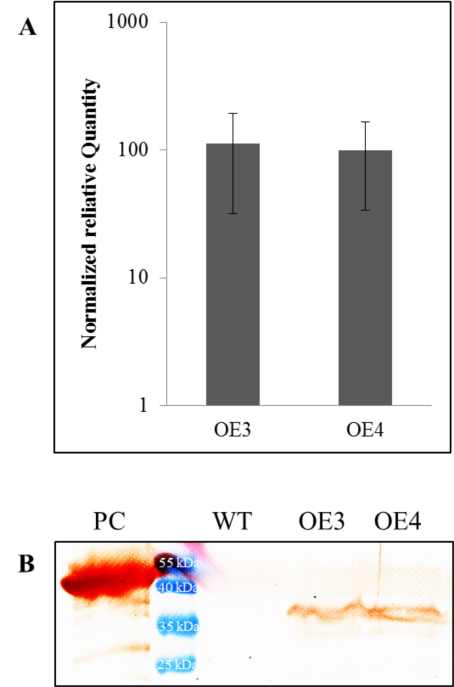


Supplementary Figure S4: (A) qPCR analyses of transcript levels in 18-day-old seedlings of ArathEULS3 OE lines. Bars represent means ±SE normalized relative ArathEULS3 expression compared to expression in WT plants. (B) Western blot of protein extracts from 3-week-old transgenic seedlings. In total 70 µg of protein was analyzed per lane. Proteins were detected with a polyclonal rabbit antibody raised against the EUL domain of ArathEULS3 protein. Recombinant ArathEULS3 protein including 6xHis-tag and C-Myc-tag was used as a positive control (PC – 38.6 kDa, expected size of native ArathEULS3 – 35.6 kDa).


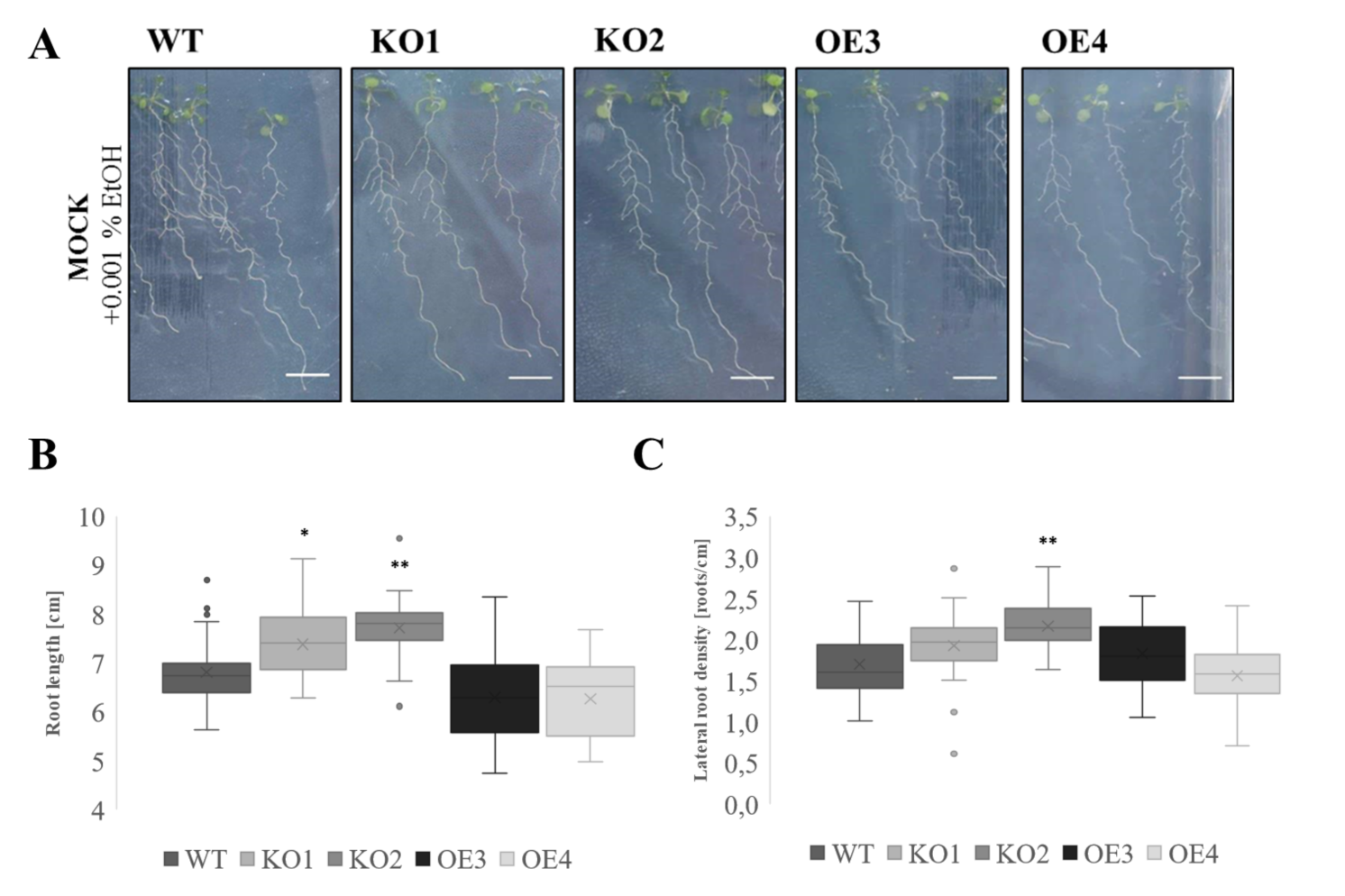
**Supplementary Figure S5:** Root phenotype, (A) root length (B) and lateral root density (C) of 10 day-old WT and transgenic lines grown on ½ MS medium supplemented with 0.001% ethanol (control for ABA treatment). The comparison between the root length of WT and transgenic lines (B) was made using an Independent-samples T-test for normally distributed data. The comparison between the lateral root density for different transgenic lines and WT (C) was made using Mann-Whitney test for not normally distributed data**.** The distributions of root length and lateral root density values are shown as box and whisker plots. The boxes represent the 25–75th percentiles, the median is indicated as a horizontal line, the mean is indicated as a cross, the outliers are represented as circles. The whiskers are equal to 1.5x the interquartile range (* p<0.05, ** p<0.01).


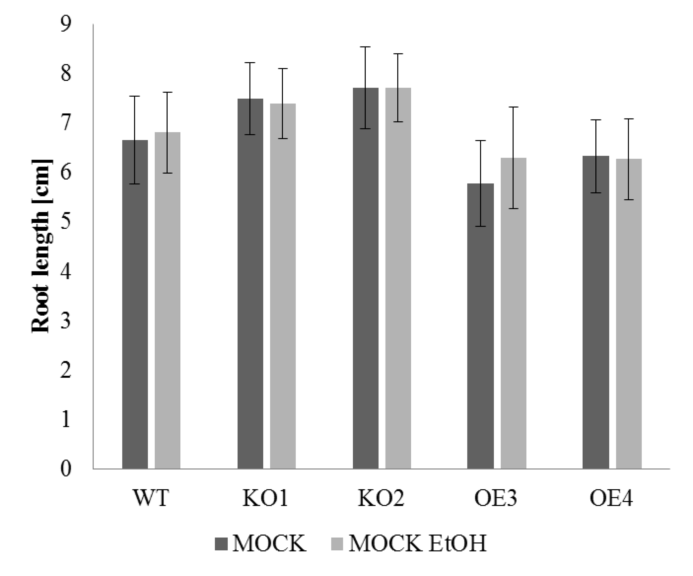


Supplementary Figure S6: Comparison between the root length for mock and mock EtOH treated samples were made by using Independent-samples T-test for normally distributed data. All bars represent means ±SD.

# Supplementary Tables

Table S3: Overview of primers used

| Target gene/sequence | Primer | 5’-3’ sequence |
| --- | --- | --- |
| Actin  (Forward primer) | evd280 | GGCTGGATTTGCTGGAGATGATGC |
| Actin  (Reverse primer) | evd281 | GTACGACCACTGGCATACAGGGA |
| Cas9  (Forward primer) | p688 | GGTGATCTCAACCCTGATAA |
| Cas9  (Reverse primer) | p689 | AAGAGCCTTGAGAAGTGTGA |
| Around mutation CRISPR1  (Forward primer) | p690 | GAGATTTTACTATGGAATAAGAAGC |
| Around mutation CRISPR1  (Reverse primer) | p691 | GTGACCAACATGACTCACAT |
| Around mutation CRISPR2  (Forward primer) | p694 | GGTCATCTCGGAAACTTGTA |
| Around mutation CRISPR2  (Reverse primer) | p695 | CCTTACCATCTCTGATCGTC |
| Sequencing CRISPR1  (Forward primer) | p692 | CGTATAGTAGACTATTCAACAAAACG |
| Sequencing CRISPR2  (Forward primer) | p696 | TCAATCCTTTGGAGTACCAC |
| Universal pEN-Chimera primer  (Reverse primer) | p624 | GCCAACTTTGTACAAGAAAGCTGGGTTAATGCC |
| Spacer-specific forward primer  CRISPR1 | p625 | GCGGCCTCTAATACGACTCACTATAGGGCGTCACCACCAACGCGACGAGTTTTAGAGCTAGAAATAGCAAG |
| Spacer-specific forward primer  CRISPR2 | p626 | GCGGCCTCTAATACGACTCACTATAGGGAGAGCCTTACTTTGAAACTCGTTTTAGAGCTAGAAATAGCAAG |
| Spacer-specific forward primer  CRISPR3  Forward outer primer for ArathEULS3 promoter sequence  Reverse outer primer for ArathEULS3 promoter sequence  Forward inner primer for ArathEULS3 promoter sequence + full attB4  Reverse inner primer for ArathEULS3 promoter sequence + full attB1r  Forward primer for sequencing middle part of ArathEULS3 promoter  Reverse primer for sequencing middle part of ArathEULS3 promoter  Forward primer kanamycin specific  Reverse primer kanamycin specific | p627  p395  p396  p393  p394  p425  p426  evd463  evd261 | GCGGCCTCTAATACGACTCACTATAGGGTAAGGTTATTCTCGCCCCAGGTTTTAGAGCTAGAAATAGCAAG  CACAACCAAAAACAAAACAAGTGG  GTAGGCGCGTAAGGATCAAAATG  GGGGACAACTTTGTATAGAAAAGTTGGTGATCGACTTTAGATTTGATATTTTC  ACTTGTAGTATTTATGTTTTGTCGATGATAC  GGTCATGAGTTGGGACCAGTG  CTAACATACTCCCTCTGTTC  GAACAAGATGGATTGCACGCAGG  TCAGAAGAACTCGTCAAGAAGGCG |

Table S4: Overview of gene specific primers used for qRT-PCR

| Target gene | Primer | 5’-3’ sequence | Primer pair efficiency |
| --- | --- | --- | --- |
| PP2A (AT1G13320) | evd727 | TCCGAGATCACATGTTCCAAACTC | 2.072±0.011 |
|  | evd728 | CCGTATCATGTTCTCCACAACCG |  |
| TIP41 (AT4G34270) | evd729 | TGAACTGGCTGACAATGGAGTG | 2.062±0.014 |
|  | evd730 | CATGAGCTTGGCATGACTCTCAC |  |
| UBC9 (AT4G27960) | evd731 | TCCTACTTCATGTAGCGCAGGAC | 2.093±0.061 |
|  | evd732 | TCCTCCAGAATAAGGGCTATCCG |  |
| ArathEULS3 (At2g39050) | evd751 | GCTGGACTCGCCGGAAGAGC | 1.892±0.019 |
|  | evd752 | TCTCGTCTTTGTACCAATGCTGTGC |  |
